# Norwalk Virus–specific Binding to Oyster Digestive Tissues

**DOI:** 10.3201/eid1206.051519

**Published:** 2006-06

**Authors:** Françoise S. Le Guyader, Fabienne Loisy, Robert L. Atmar, Anne M. Hutson, Mary K. Estes, Nathalie Ruvoën-Clouet, Monique Pommepuy, Jacques Le Pendu

**Affiliations:** * Institut Français de Recherche pour l'Exploitation de la Mer, Nantes, France;; † Baylor College of Medicine, Houston, Texas, USA;; ‡ Ecole Nationale Vétérinaire, Nantes, France;; § Institut National de la Santé et de la Recherche Médicale, Nantes, France;; ¶ Université de Nantes, Nantes, France

**Keywords:** Norovirus, calicivirus, shellfish, carbohydrate, pathogen selection, virus-like particles, research

## Abstract

The primary pathogens related to shellfishborne gastroenteritis outbreaks are noroviruses. These viruses show persistence in oysters, which suggests an active mechanism of virus concentration. We investigated whether Norwalk virus or viruslike particles bind specifically to oyster tissues after bioaccumulation or addition to tissue sections. Since noroviruses attach to carbohydrates of the histo-blood group family, tests using immunohistochemical analysis were performed to evaluate specific binding of virus or viruslike particles to oyster tissues through these ligands. Viral particles bind specifically to digestive ducts (midgut, main and secondary ducts, and tubules) by carbohydrate structures with a terminal N-acetylgalactosamine residue in an α linkage (same binding site used for recognition of human histo-blood group antigens). These data show that the oyster can selectively concentrate a human pathogen and that conventional depuration will not eliminate noroviruses from oyster tissue.

## Abstract 2

Specific binding of virus to oysters can selectively concentrate a human pathogen.

Twelve years ago, the question, "Should shellfish be purified before public consumption?" was asked in Lancet (*1*). Since then, new evidence of gastroenteritis outbreaks linked to shellfish consumption, even depurated shellfish, has been published, and raw or cooked oysters are the predominant bivalve mollusks involved (*2**–**5*). Regulations for *Escherichia coli* counts in shellfish-growing waters (United States) or shellfish meat (European Community) have failed to protect consumers because most shellfish-associated gastroenteritis outbreaks have a viral origin (*4*). Enteric viruses are different from enteric bacteria in terms of resistance to sewage treatment, persistence under unfavorable conditions such as occur in sea water, and transmission into the environment (*6**–**8*). Shellfish mollusks cultivated in coastal areas close to human activities can be contaminated by human sewage, which can spread >100 types of viruses (*9*). Viruses persist in shellfish for an extended period and can adversely affect public health; despite improvements, depuration does not eliminate viral particles (*2**,**10**–**14*).

Noroviruses are the most frequent cause of diarrhea outbreaks in all age groups (*8**,**15*). These viruses, which are commonly associated with foodborne and waterborne outbreaks, are resistant to sewage treatment and are present in high concentrations during the epidemic season (*3**,**7**,**15*). They are the primary pathogens associated with shellfishborne outbreaks worldwide (*3**,**4*). Oysters are rapidly contaminated, as shown by outbreaks linked to accidental input, and viruses then persist for up to several weeks (*2**,**13**,**16*). These data suggest that contamination by a passive process during mollusk filter feeding may be too simplistic an explanation. We tested whether oysters can actively capture noroviruses and determined the fate of prototype genogroup I Norwalk virus particles in bioaccumulation experiments. Since noroviruses can attach to carbohydrates of the ABH and Lewis histo-blood group family in humans, we also examined the possibility of a similar specific binding to oyster tissues through related carbohydrates (*17**,**18*).

## Materials and Methods

### Norwalk Virus Strain and Recombinant Viruslike Particles

Norwalk virus strain 8FIIa was purified from a stool sample of an infected volunteer and assayed by reverse transcription–polymerase chain reaction (RT-PCR) endpoint dilution (RT-PCR units) to determine the amount of viral RNA in the sample (*19*). Based on the equivalence of 1 RT-PCR unit to 30–40 genomic copies of RNA, the titer was estimated to be ≈3 × 10^10^ particles/mL (*2*).

Constructs containing open reading frames 2 and 3 were used to produce recombinant viruslike particles (VLPs) as described previously (*20*). Virus concentration, as calculated with the detergent compatible protein assay (Bio-Rad Laboratories, Hercules, CA, USA), was 2.11 × 10^15^ particles/mL. The number of particles in 1 μg of VLPs was calculated by multiplying the molecular mass of the major capsid protein VP1 (58 kDa) by the number of copies of the protein in a particle (180) and by a mass of 1 Da (1.66 × 10^–24^ g) (*21*).

Mutants in the P2 subdomain of VP1 were generated by Ala point substitution and used to produce recombinant VLPs His 329 (H329A), Asn 331 (N331A), and Trp 375 (W375A) (*22*). Concentrations of these 3 mutant VLPs were adjusted to 5.78 × 10^14^ particles/mL.

### Oysters

All experiments were performed with *Crassostrea gigas* from a clean area (Class A, European Regulations), with no viral contamination, as tested after viral concentration, RNA extraction, and RT-PCR (*2*). Two batches of oysters collected in March and October 2004 were used to prepare tissue sections.

### Bioaccumulation of Norwalk Virus and Recombinant VLPs

Norwalk virus (5 × 10^8^ particles) or recombinant VLPs (10^12^ or 10^9^ particles) were added to 500 mL clean sea water and homogenized for 5 min. Three oysters were added to the sea water and incubated at room temperature for 12 or 24 h. Sea water was continuously aerated to maintain adequate oxygen levels. A negative control was used under similar conditions but without the addition of Norwalk virus or recombinant VLPs.

### Immunohistochemical Analysis

Oysters (uncontaminated or after bioaccumulation) were shucked, and the flesh was fixed in 10% formaldehyde for 48 hours. The digestive gland was then dissected, embedded in paraffin, and sliced into thin sections (5 μm). After preparation of tissue sections, sections from uncontaminated oysters were covered with 4 × 10^9^ particles of recombinant wild-type VLPs, 3 × 10^9^ particles of Norwalk virus, or 2 × 10^8^ particles of mutant VLPs, incubated overnight at 4°C, and washed 3 times (5 min per wash) in phosphate-buffered saline (PBS) at room temperature (*23*). VLPs bound to oyster tissue or virus trapped after the bioaccumulation experiments was detected by using a rabbit polyclonal antibody to recombinant VLP as previously described (*23*). Negative controls included sections from uncontaminated samples not exposed to Norwalk virus or recombinant VLPs and virus-exposed sections without antibody. Immunoreactivity, which was detected by microscopic analysis, was graded as strong (intense brown-red staining), weak (pale brown-red staining), or negative (no staining).

### Inhibition of Recombinant VLP Binding to Tissue Sections

Treatment with periodate was performed as previously described (*23*). VLPs were incubated overnight on slides, and the immunohistochemical detection was conducted. Saliva samples from 18 persons with secretor type O, 14 with secretor type A, 4 with secretor type B, and 5 nonsecretors were tested. Phenotyping was performed by enzyme-linked immunosorbent assay, and secretor phenotype was confirmed by genotyping as previously described (*17*). For the inhibition assay, VLPs were mixed with saliva samples diluted 1:100 for 90 min at room temperature, placed on shellfish tissues, and incubated overnight at 4°C. Positive (without treatment) and negative (without recombinant VLPs) samples were included in this assay.

### Binding and Inhibition by Specific Lectins and Carbohydrate-specific Antibodies

Three biotinylated lectins, derived from *Helix pomatia* (HPA), *Dolichos biflorus* (DBA), and *Ulex europaeus* (UEA-1) (Biovalley SA, Marne la Vallée, France), were used for analysis of binding to tissues and inhibition of VLP binding. UEA-1 recognizes the H type 2 trisaccharide and shows some cross-reactivity with Le^y^. HPA and DBA recognize α-linked N-acetylgalactosamine residues. For the binding assay, lectins were applied to shellfish tissues for 30 min at different concentrations (50, 20, 10, 5, and 1 μ>g/mL).

Monoclonal antibodies were used for tissue staining. The antibodies used were anti-A (3-3A), which recognizes all A epitopes; anti-A types 3/4 (III-2A3, III-2A18, and III-2A24); anti-A type 2 (III-2A5); anti-H/Le^b^ (LM-137); anti-H type 2 (19-OLE); anti-Le^y^ (12-4LE); and anti-H type 1. The primary antibodies were serially diluted, incubated with tissues overnight at 4°C, and subjected to standard immunohistochemical analysis (*23*). Negative (without lectin or primary antibody) and positive (known rat or human tissue sections) controls were also tested. For inhibition assays, antibodies or lectins were applied to shellfish tissues for 1 h at 37°C or 1 h at 4°C, respectively, at concentrations 10-fold higher than the lowest dilution that showed binding to the oyster tissues, incubated with VLPs, and analyzed as described above.

## Results

### Binding of Virus in Oysters after Bioaccumulation

Oysters were immersed in sea water seeded with Norwalk virus at a final concentration of 10^6^ particles/mL or recombinant VLPs at final concentrations of 2 × 10^9^ and 2 × 10^6^ particles/mL. After incubation for 12 h, virus was detected in digestive diverticula. Immunostaining showed particles in the lumen of tubules and ducts or in phagocytes between epithelial cells and in the surrounding connective tissue (Figure, panels A and B). No difference was observed for the 2 recombinant VLP concentrations used. After incubation for 24 h in sea water at final concentrations of 5 × 10^8^ and 5 × 10^9^ particles/mL for Norwalk virus and recombinant VLPs, respectively, similar results were obtained with particles binding to digestive ducts and isolated cells in connective tissue (data not shown).

**Figure:**
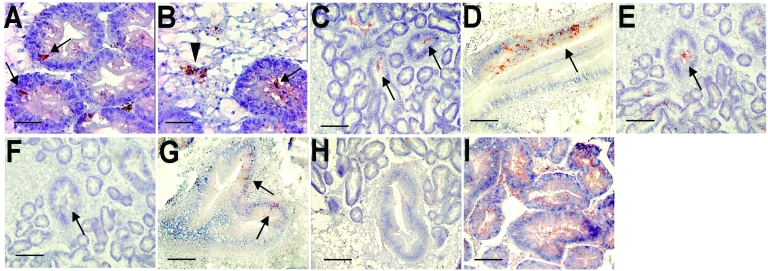
Immunohistochemical detection of Norwalk viruslike particles (VLPs) in oyster digestive tissue. A) VLPs in the digestive diverticulum 12 h after seeding sea water with 10^9^ particles. The arrows show immunoreactivity detected in intraepithelial cells. B) VLPs in the digestive diverticulum 12 h after seeding sea water with 10^12^ particles. The arrowhead shows immunoreactivity in a phagocyte located in connective tissue, and the arrow shows immunoreactivity in the lumen of a digestive tubule. C) Attachment of recombinant VLPs to secondary ducts of the digestive diverticula after incubation on tissue sections. The arrows show immunoreactivity in the lumen of ducts. D) Attachment of Norwalk virus to a main digestive duct. The arrow shows immunoreactivity in epithelial duct cells. E and F) Attachment (arrows) of recombinant VLPs to digestive ducts without (E) or with (F) periodate treatment of serial tissue sections. G) Binding of VLPs (arrows) to a main digestive duct of VLPs from the H331A mutant capsid protein. H) Lack of binding of the H329A mutant. I) Binding of HPA lectin to the digestive diverticula. Scale bars: A and B, 10 μm; C, E, F, and H, 40 μm; D, G, and I, 20 μm.

### Binding of Virus in Oyster Digestive Tissues

The binding of virus or recombinant VLPs was tested directly on oyster gut sections. Both recombinant VLPs and native virions bound to the midgut, the main and secondary ducts of the digestive diverticula, and tubules, although labeling of tubules was weaker (Figure, panels C, D, and E). Binding to connective tissue was not observed. Staining was not observed in negative controls.

Recombinant VLPs can attach to human digestive epithelial cells by recognizing carbohydrate structures. To determine if attachment to oyster digestive cells was also carbohydrate dependent, tissues sections were treated with 10 mmol/L sodium periodate before adding the VLPs. A reduction in VLP binding occurred, which suggested that, similar to the case with human tissue, binding to oyster tissue involves carbohydrates (Figure, panels E and F).

### Inhibition of Virus Binding to Oyster Digestive Cells by Human Saliva

Since attachment of recombinant VLPs to human tissue sections involves carbohydrates of the ABH, secretor, and Lewis histo-blood group family, which are present on human salivary mucins, we evaluated the ability of saliva of different ABO and secretor phenotypes to block the binding of VLPs to shellfish tissues. Complete inhibition of binding was observed after pretreatment of the recombinant VLPs with type A secretor saliva, and binding was markedly reduced after incubation with type O secretor saliva. However, after incubation with type B secretor saliva or nonsecretor saliva, binding was as strong as that observed in positive controls (Table 1). All samples in subgroups of saliva showed similar patterns.

**Table 1:** Inhibition of binding of Norwalk viruslike particles (VLPs) to oyster digestive tissues by saliva

Saliva	No. samples tested	VLP binding*
A	14	–
O	18	+
B	4	+++
Nonsecretor	5	+++
Control	5	+++

*After incubation with saliva, no (–), weak (+), or strong (+++) VLP binding to oyster digestive tissues was observed. For controls, VLPs were applied to shellfish tissues in phosphate-buffered saline/1% bovine serum albumin instead of a saliva sample diluted in the same buffer.

### Binding of Mutant Recombinant VLPs

Our results suggested that attachment of VLPs to oyster tissue involves carbohydrate binding sites that overlap the site that attaches to human digestive cells. This site has been mapped to a restricted area of the viral capsid P2 domain. Mutations in key residues in this domain inactivate the binding activity to histo-blood group structures. To confirm that the binding to oyster tissue involved the same binding site, VLPs with point mutations were tested. Among 3 mutants, only mutant N331A binds to tissues similar to parental recombinant VLP (Figure, panel G). The 2 other mutants, H329A and W375A, did not bind to tissues (Figure, panel H).

### Inhibition of VLP Binding by Antibodies to Carbohydrate and Lectins

Antibodies to carbohydrates on cells in the human digestive tract can inhibit binding of recombinant VLPs to human tissue (*18*). Primary antibodies that recognize various determinants of the histo-blood group family were evaluated for binding to oyster digestive tract and inhibition of VLP binding. Antibodies that recognize all types of A determinants (3-3A) or those that are restricted to A type 3/4 determinants bound strongly to shellfish tissues (Table 2). Anti-H type 1 BG-4 labeled oyster digestive cells intracellularly and also labeled connective tissue. Antibody LM137, which recognizes all H determinants and Le^b^ tetrasaccharide, also labeled connective tissue and digestive epithelial cells intracellularly. However, staining had a more punctiform appearance. Despite recognition of oyster ligands, in inhibition assays none of these antibodies inhibited binding of VLPs to oyster tissues. Antibodies directed against H type 2 (19-OLE,), Le^y^ (12-4LE), or A type 2 epitopes (III-2A-5) showed little staining of shellfish tissues and when positive, staining did not correspond to cells to which VLPs bind. These results indicate that oyster tissues have carbohydrates that resemble human histo-blood group antigens, but structures recognized by VLPs were not identical to those recognized by these antibodies.

**Table 2:** Binding of carbohydrate antibodies to oyster digestive tissues

Antibody	Binding*	Tissue
Anti-A all types	+++	Digestive cells
Anti-A types 2	+	
Anti-A type 3/4	+++	Digestive cells
Anti-H type 1	+++	Digestive cells (intracellularly) and connective tissue
Anti-H type 2	+	
Anti-H/Le^b^	+++	Digestive cells (intracellularly) and connective tissue
Anti-Le^y^	+	

*+++, strong; +, weak.

Lectins have been used to determine the distribution of carbohydrate residues in tissue. Since lectins have a broader reactivity than monoclonal antibodies, we used lectins to identify recombinant VLP-specific carbohydrate ligand in oyster tissue. UEA-1 bound to shellfish tissues, but only at high concentration (50 μg/mL), whereas 2 other lectins, DBA and HPA, bound at a lower concentration (1 μg/mL) (Table 3). Tissue and cellular distribution of staining overlapped with that of the recombinant VLPs (Figure, panel I). When these lectins were used in an inhibition assay, only HPA had an inhibitory effect on binding of recombinant VLPs to shellfish tissues. Complete inhibition was observed at a concentration of 25 μg/mL (Table 3). This inhibitory effect was reproducible and observed on tissues from different shellfish, indicating that attachment of recombinant VLPs to oysters involves carbohydrate structures with a terminal N-acetylgalactosamine residue in an α linkage.

**Table 3:** Lectin binding to oyster digestive tissues and inhibitory effect on binding of Norwalk viruslike particles (VLPs)

Lectin*	Binding (μg/mL)†	VLP inhibition (μg/mL)‡
DBA	1	None
UEA-1	50	None
HPA	1	25

*Derived from *Dolichos biflorus* (DBA), *Ulex europaeus* (UEA-1), and *Helix pomatia* (HPA). †Lowest concentration showing binding to digestive tissues. ‡Lowest dilution showing a complete inhibition of VLP binding to digestive tissues. None indicates no inhibition at 50 μg/mL.

## Discussion

Virus-mediated disease can be transmitted when contaminated shellfish are eaten. Oysters are believed to act as filters or ionic traps, passively concentrating particles. A simple depuration process should be sufficient to rid oysters of virus, as observed for bacteria (*12*). However, long-term virus persistence in shellfish is a serious public health issue, and depuration or relaying is known to be inefficient (*10**,**12**,**14*). After bioaccumulation, only 7% of Norwalk virus is depurated, compared to a 95% reduction in bacterial levels (*13*). Virus is located mainly in pancreatic tissue (digestive diverticula), and various mechanisms have been suggested to explain differences between oyster species regarding virus accumulation, such as mechanical entrapment or ionic bonding (*10**,**13**,**24**,**25*).

Our data demonstrate specific binding of viral particles from a genogroup I norovirus to the oyster digestive tract and suggest a specific mechanism for concentration of virus particles. We tested recombinant VLPs of prototype genogroup I Norwalk virus. VLPs are stable in the marine environment and the disinfection processes (*14**,**26*). Bioaccumulation and tissue-binding experiments showed no difference between native Norwalk virus and VLPs, which confirmed that VLPs are good surrogates of infectious virions.

Different results were seen between bioaccumulation experiments and particle binding to tissues sections. After bioaccumulation, some viral particles were detected in phagocytes in either epithelium or connective tissue. This finding could reflect elimination of virus during digestion. The time required for food to pass through the entire shellfish intestinal tract varies from 90 to 150 min. We do not know whether immunoreactive material detected in phagocytes corresponds to particle degradation and digestion or if particles can escape digestion. Binding to main ducts may provide a mechanism for viral particles to avoid entering the digestive system and being degraded. Specific attachment of virus to oyster cells and capture by phagocytes may explain why depuration in oysters is not an effective mechanism for eliminating virus.

Virus accumulation in oysters may depend on factors such as water temperature, mucus production, glycogen content of connective tissue, or gonadal development (*25*). In our study, no difference was seen in binding location between samples collected in March or October, although studies during other seasons are warranted.

Human susceptibility factors for norovirus infections that depend upon carbohydrates of the ABH, secretor, and Lewis histo-blood group family have been observed (*17**,**27*). We showed that recognition of oyster digestive epithelial cells by recombinant VLPs also involves carbohydrates. The ability of human saliva to inhibit attachment of VLPs to oyster tissue in a histo-blood group–dependent manner indicates involvement of the histo-blood group binding site and the viral P2 subdomain (*18*).

Similar to human histo-blood group structures, mutant VLPs showed that an alanine substitution at positions H329A and W375A prevented binding to oyster tissue. However, a mutant with a substitution at position N331A did not affect binding. This binding specificity identified the amino acids required for binding and further confirmed the similarity with the mechanism of recognition of human tissues (*22*). However, inhibition experiments using antibodies to carbohydrates showed that ligands on oyster tissues are not identical to those on human tissues. Since attachment of recombinant VLPs was blocked by lectin from *H*. *pomatia*, which recognizes α-linked N-acetylgalactosamine terminal residues of glycans, the oyster ligands are similar to those of histo-blood group A. The DBA lectin did not inhibit attachment of recombinant VLPs, which indicates that it does not bind oligosaccharide structures recognized by VLPs and HPA. Although DBA binds to N-acetylgalactosamine residues similar to HPA, the 2 lectins have different carbohydrate specificities that depend on the subjacent sugar residues (*28*). Thus, Norwalk virus binds to oyster tissues through an A-like carbohydrate structure recognized by HPA at a binding site also used for attachment to carbohydrate on human epithelial cells.

Genogroup I and II strains of norovirus show various binding patterns with different carbohydrate structures of the histo-blood group family, which suggests coevolution of this group of viruses and their host or carrier vector. The ability of Norwalk virus to bind to oysters tissues at the same binding site as that used to bind to human tissues suggests a possible coevolution mechanism involving the oyster as an intermediary vector. This mechanism would favor selection of some viruses, such as Norwalk virus, over other viruses that are unable to bind to the oyster and would not be transmittable by this intermediary host. Epidemiologic data suggest a predominance of genogroup I strains in oyster-related gastroenteritis outbreaks, whereas genogroup II strains are predominant in other food-related outbreaks (*2**,**3**,**5**,**8**,**15**,**16*). To clearly address this point, binding of other norovirus strains needs to be evaluated because VLP-carbohydrate binding patterns differ, and binding should also be evaluated with other shellfish species (*18**,**27*).

As knowledge increases in understanding the binding of these viruses in humans, we will likely further understand more about their behavior in shellfish. This knowledge may help identify species that could be less sensitive to contamination. Since the oyster can actively and specifically bind a human pathogen, this knowledge has practical consequences because conventional depuration cannot eliminate noroviruses from oyster tissues.
